# Combined Use of Digital and Analog Physical Therapy in Patients With Musculoskeletal Disorders and Indicators of Chronicity: German Claims Data Analysis

**DOI:** 10.2196/63935

**Published:** 2025-06-09

**Authors:** Silke Frey, Annika Schmitz, Udo Schneider, Linda Kerkemeyer, Birgitta Weltermann

**Affiliations:** 1 Institute of General Practice and Family Medicine, University Hospital Bonn, University of Bonn, Venusberg-Campus 1, Bonn, 53127, Germany, +49 228 287 11156; 2 Techniker Krankenkasse, Hamburg, Germany; 3 LiKe Healthcare Research GmbH, Berlin, Germany

**Keywords:** claims analysis, digital health interventions, digital health applications, DiGA, mHealth, utilization, musculoskeletal disorders, physical therapy, exercise therapy, chronicity

## Abstract

**Background:**

Musculoskeletal disorders are highly prevalent worldwide and contribute significantly to the overall burden of disease. Regular physical therapy with trained physiotherapists is recommended in the guidelines. Recently, digital physical therapy offered by digital health interventions was shown to be effective. However, the evidence on its real-world usage in health care systems is limited.

**Objective:**

Based on claims data, this study examined the current usage of digital health applications (DiGAs) for musculoskeletal disorders in the German health care system. Patients with standalone digital physical therapy were compared to those with a combination of analog and digital physical therapy. In addition, predictors for concomitant use were identified.

**Methods:**

This retrospective cohort study analyzed claims data from Germany’s largest statutory health insurance. Patients who used DiGA for musculoskeletal disorders at least once were included. Sociodemographic and medical characteristics of patients receiving standalone and concomitant physical therapy were compared. Statistical analyses comprised univariate analyses and binomial logistic regression.

**Results:**

Of the 6090 individuals, 58.2% (3543/6090) were prescribed physical therapy within 6 months before or after DiGA prescription. In this population, 36.3% (2210/6090) used DiGA and analog physical therapy at the same time. Concomitant physical therapy was significantly more likely in patients with chronicity risk (odds ratio [OR] 1.49, 95% CI 1.31‐1.69; *P*<.001) or established chronicity (OR 2.76, 95% CI 2.22‐3.47; *P*<.001), female gender (OR 1.48, 95% CI 1.33‐1.66; *P*<.001), and higher age (OR 1.02, 95% CI 1.02‐1.02; *P*<.001).

**Conclusions:**

The findings highlight the diverse utilization patterns of DiGAs among patients with musculoskeletal disorders. Chronicity emerged as an important predictor for combined digital and analog physical therapy. These findings support considerations on integrating digital health interventions into current guidelines.

## Introduction

Musculoskeletal disorders (MSDs), back pain in particular, contribute to disability, with approximately 1.71 billion people affected worldwide [1]. The burden of MSDs has increased significantly over recent decades, resulting in 107,885,832.6 disability-adjusted life years in 2015 globally [2]. In Europe, the burden of MSDs is even higher than in other continents [2]. The prevalences of lower back pain and neck pain in Germany reached 61.3% and 45.7% over a 12-month period, with women being significantly more often affected than men [3]. Similarly, 17.3% of women and 15.1% of men aged 18-79 years experienced knee pain within the last 24 hours in a German random sample (N=8151) [4]. The increasing incidence of MSDs has resulted in considerable economic costs for individuals and society due to lost workdays and conservative or surgical interventions [2,5].

Exercise therapy is effective in reducing pain and enhancing functionality in individuals suffering from MSDs, including chronic lower back pain, knee osteoarthritis, and fibromyalgia. Due to its demonstrated benefits, this approach is recommended in medical guidelines and reviews [6-8]. Recently, digital health interventions (DHIs) have become a new therapeutic option to guide patients in regular physical activity. DHIs cover various medical conditions and offer features such as symptom tracking, educational content, video-guided therapeutic exercises, and feedback. The following textbox displays the content and therapeutic approaches of digital and analog physical therapy in comparison (Textbox 1).

Textbox 1. **Comparison of digital and analog physical therapy.**

**Digital physical therapy - digital health intervention (DHI)**

- Structured exercise programs with video, audio, or app-based guidance
- Algorithm-driven progression and adaptation
- Automated reminders & frequent exercise sessions
- Monitoring of pain levels and physical functionality based on patient-reported outcome measures (PROMs) and self-tests
- Educational content

The content of DHIs is based on information in the DiGA directory of the Federal Institute for Drugs and Medical Devices, Germany [9].

**Analog physical therapy**

- Supervised exercise therapy with individual feedback and correction
- Therapist-guided progression and adaptation
- Scheduled in-person sessions
- Initial assessment & monitoring of pain levels and physical functionality documented by the therapist
- Patient education and home exercises

The content of analog physical therapy is presented as published in the German Remedy Guideline (Helmittel-Richtlinie) [10].

Analog physical therapy provides direct professional supervision and individualized feedback, but is resource-intensive and limited to scheduled sessions. In contrast, digital physical therapy facilitates frequent exercises independent of time and location, but relies on patients’ self-motivation and adherence. Both approaches offer distinct advantages and challenges, making them suitable for different patient populations.

Evidence indicates that DHIs are effective in managing MSDs. A recent systematic review by Nagel and colleagues [11], which included 16 high-quality, randomized controlled trials with a total of 1840 patients with MSDs, confirmed that digital physical therapy reduces back pain significantly. There was moderate or limited evidence for positive effects on other body parts affected by MSDs [11]. These findings are in line with previous studies confirming the efficacy of DHIs in treating MSDs [12-14]. Furthermore, a systematic review and meta-analysis by Fatoye and colleagues [15], referring to 10 studies from 6 countries, concluded that DHIs are cost-effective for people affected by MSDs.

Because of the promising effects of DHIs and the ongoing digital transformation of the health care sector, Germany introduced so-called digital health applications (*Digitale Gesundheitsanwendung* [DiGA]) in 2020. These software-based medical devices are prescribed by physicians and psychotherapists and are reimbursed by statutory health insurances (SHIs). Patients with a confirmed diagnosis can apply for an activation code for a DiGA through their SHI, even in the absence of a prescription (self-application). According to the Digital Healthcare Act (*Digitale-Versorgung-Gesetz* [DVG]), each DiGA is evaluated and gets a (temporal) approval by the Federal Institute for Drugs and Medical Devices (*Bundesinstitut für Arzneimittel und Medizinprodukte* [BfArM]). This fast-track process ensures compliance with quality standards, such as product safety, data protection and user-friendliness, and assesses health care efficacy. Currently, nearly 60 DiGAs are listed in the directory of the BfArM, with MSDs being a significant focus in addition to mental illnesses [16]. The number of DiGA users has increased concurrently with the growing number of prescribable DiGAs, reaching a total of 374,377 individuals by September 2023 [17].

Despite the growing interest in DiGAs, their utilization currently falls short of expectations. This disparity arises from health care providers’ skepticism concerning factors such as the efficacy and data security of DiGAs. In addition, DiGAs are not yet part of medical guidelines [18]. In general, DiGAs can be used as substitutes, as a complementary add-on to existing health care services, or as partial replacements, for example, to bridge delays in health care provision, such as waiting times for physical therapy [19].

Previous publications from authorities, interest groups, or SHI companies have primarily examined and described the use of DiGAs in health care [17,20-22]. However, there are no comparisons to analog therapies nor tracking of individual patient journeys in a real-world setting. Therefore, this study aimed to analyze the status quo of DiGA utilization for MSDs based on claims data, with a focus on comparing standalone digital therapy to concomitant digital and analog physical therapy.

## Methods

### Study Design and Cohort

A retrospective cohort study analyzing DiGA users with MSDs was conducted. Nationwide claims data of the largest sickness fund in Germany, the Techniker Krankenkasse (TK), covering approximately 11 million people, were provided in pseudonymized form. All adults (at least 18 y) insured by TK who received and activated at least one DiGA for MSD (Vivira [Vivira Health Lab GmbH], Companion patella [PrehApp GmbH], or Mawendo [Mawendo GmbH]) since September 2020 were included in the study. Eligible patients were identified via the pharmaceutical central number of the DiGAs.

Given delays of up to 9 months between the occurrence, processing, and availability of claims data especially for outpatient care, services provided until March 31, 2023, were analyzed. In addition, each patient’s claims data required a minimum of 6 months of follow-up, restricting inclusion to patients prescribed a DiGA before October 1, 2022. At the time, three DiGAs for MSDs were listed in the DiGA directory: Vivira for unspecific lower back pain and osteochondrosis, as well as Companion patella and Mawendo for anterior or patella-related knee pain. Detailed descriptions of the functionalities of these DiGAs are presented in the Multimedia Appendix 1. During the observation period, patients had to be insured continuously with TK, with a tolerated gap of up to 30 days. Data of individuals with a privacy protection indicator were not transferred; these included persons who objected to the use of their data for research purposes as well as employees of the TK.

Using either the DiGA prescription date or the receipt of the self-application at TK, a tailored preobservation and follow-up period were determined for each patient. Each patient-individual quarter was defined by 90 days, the duration of the DiGA prescription. The preobservation comprised 8 quarters before the DiGA prescription, while the follow-up ideally encompassed eight quarters, with a minimum of 2. The analysis included people who died during the follow-up.

### Operationalization of Physical Therapy and Chronicity

The sample was divided into 2 groups based on the utilization of physical therapy in addition to DiGA. The group with DiGA and analog physical therapy comprised patients who received at least one physical therapy session within the last 2 preobservation quarters and the first 2 follow-up quarters, while the group receiving DiGA as standalone therapy comprised patients who did not receive physical therapy within the same time frame.

Additional subgroups were created based on the degree of chronicity, using proxies such as sick leave days and medication patterns due to the absence of clinical information in claims data. This approach follows the methodology of the claims data analysis on back pain and the significance of chronicity by Freytag and colleagues [23], categorizing individuals as follows:

1. Those incapacitated for more than 42 days in the last 2 preobservation and the first 2 follow-up quarters or who received opioid prescriptions at least twice were classified as “with indication of chronicity.”
2. Insured individuals were included in the “with indication of chronicity risk” group if the outpatient diagnoses included at least one of the following psychological comorbidities in the last 2 preobservation and the first 2 follow-up quarters: F32* (depressive episode), F33* (recurrent depressive disorder), F34.1 (dysthymia), F34.8 (other persistent affective disorder), F34.9 (unspecified persistent affective disorder), F38* (other affective disorder), F41.2 (mixed anxiety and depressive disorder), F45.4 (persistent somatoform pain disorder), F48.0 (neurasthenia), F43.20 (adjustment disorder), F43.21 (adjustment disorder with prolonged depressive reaction), F43.22 (adjustment disorder with anxiety and depressive reaction), F54 (psychological behavioral factors associated with disorders or diseases classified elsewhere), F62.80 (personality change due to chronic pain syndrome).
3. All other insured individuals who did not fall into the above-mentioned groups were categorized as “without indication of chronicity.”

### Ethical Considerations

All methods conducted in this study followed relevant guidelines and regulations, including the German Reporting Standard for Secondary Data Analyses (*Standardisierte Berichtsroutine für Sekundärdaten Analysen* [STROSA]) and Good Practice in Secondary Data Analysis (*Gute Praxis Sekundärdatenanalyse* [GPS]) guidelines. The data were transmitted with permission of the German Federal Office for Social Security under § 75 of the Social Code (*Zehntes Buch Sozialgesetzbuch - SGB X*). The Ethics Committee of the Medical Faculty at the University of Bonn has reviewed the study proposal and raised no ethical or legal concerns (054/23-EP). The data protection officer of the University Hospital Bonn actively contributed to the data protection concept and provided a positive assessment of the project. Individual informed consent for data usage was not required, and no experiments or surveys were conducted to gather the data.

### Data Analysis

Plausibility and validity checks were performed for all variables in the datasets. Subsequently, the individual preobservation and follow-up were determined for each insured person. Descriptive analyses were conducted for the entire cohort as well as for the physical therapy subgroups. Absolute and relative frequencies are reported for categorical variables. Differences between physical therapy groups were assessed using the *χ*^2^ test or Fisher exact test. The visualization of all patient pathways, the 10 most common pathways, and the state distribution in each quarter was created using the TraMineR package for sequence analysis in R [24].

A binomial logistic regression analysis was performed to investigate the potential factors associated with using DiGA as a standalone therapy or in conjunction with physical therapy. Chronicity risk, age, gender, and DiGA characteristics were incorporated into the model via a forward variable selection process, considering the significance of added variables, the Akaike information criterion, and Nagelkerke R². Odds ratios (ORs), along with their corresponding 95% CI and *P* values, were computed to evaluate the presence and strength of the associations between the covariates and the utilization of physical therapy. Multicollinearity in the final model was assessed by calculating the variance inflation factor. The significance level was predetermined at 5%. All analyses were conducted using R Studio (Posit, PBC) [25].

## Results

### Sociodemographic Characteristics

After applying inclusion and exclusion criteria, 6090 patients with at least one DiGA for MSD were eligible for the analysis. Within the last 2 preobservation quarters, the DiGA quarter itself, and the subsequent quarter, 3543 individuals (3543/6090, 58.2%) received at least one physical therapy prescription. For 36.3% (2210/6090) of the study population, there was an overlap in the actual duration of physical therapy and the usage of DiGA. Except for the number of patients who died during follow-up, the highest professional training, and the membership of the individuals, all other sociodemographic and medical characteristics differed significantly between patients who did and did not receive physical therapy (Tables1  2). For instance, the proportion of women was greater among patients receiving physical therapy (*χ*^2^_1_=75.1; *P*<.001), and these patients were significantly older (*χ*^2^_6_=166.1; *P*<.001). Further details regarding sociodemographic characteristics can be found in Table 1.

**Table 1.** Sociodemographic characteristics of the study population.

Item and category	Total, n (%)	Without physical therapy, n (%)	With physical therapy, n (%)	*χ*2 (*df*)	*P* value
All patients	6090 (100)	2547 (41.8)	3543 (58.2)	—^a^	—
Gender				75.1 (1)	<.001
Women	3970 (65.2)	1501 (58.9)	2469 (69.7)		
Men	2120 (34.8)	1046 (41.1)	1074 (30.3)		
Age, years				166.1 (6)	<.001
15 to <20	60 (1)	35 (1.4)	25 (0.7)		
20 to <30	771 (12.7)	442 (17.4)	329 (9.3)		
30 to <40	1406 (23.1)	658 (25.8)	748 (21.1)		
40 to <50	1219 (20)	517 (20.3)	702 (19.8)		
50 to <60	1604 (26.3)	562 (22.1)	1042 (29.4)		
60 to <65	530 (8.7)	168 (6.6)	362 (10.2)		
65 to >65	500 (8.2)	165 (6.5)	335 (9.5)		
Death				0.0 (1)	.99, >.99^b^
Died during follow-up	6 (0.1)	2 (0.1)	4 (0.1)		
Membership				4.3 (1)	.04
Individual member	5662 (93)	2347 (92.1)	3315 (93.6)		
Family insured member	428 (7)	200 (7.9)	228 (6.4)		
Highest professional training				12.3 (6)	.06
No professional qualification	196 (3.2)	93 (3.7)	103 (2.9)		
Vocational training	1839 (30.2)	743 (29.2)	1096 (30.9)		
Bachelor’s degree	304 (5)	147 (5.8)	157 (4.4)		
Master’s degree	1119 (18.4)	482 (18.9)	637 (18)		
Technician or master	203 (3.3)	90 (3.5)	113 (3.2)		
Doctorate	101 (1.7)	48 (1.9)	101 (2.9)		
N/A^c^	2328 (38.2)	944 (37.1)	1336 (37.7)		
Domicile				17.6 (3)	<.001
City (≥100,000 inhabitants)	2742 (45)	1198 (47)	1544 (43.6)		
Urban counties (≥150 inhabitants per km^2^)	2359 (38.7)	988 (38.8)	1371 (38.7)		
Densely populated rural counties (100‐150 inhabitants per km^2^)	589 (9.7)	222 (8.7)	367 (10.4)		
Sparsely populated rural counties (<100 inhabitants per km^2^)	395 (6.5)	135 (5.3)	260 (7.3)		
N/A	5 (0.1)	4 (0.2)	1 (0)		

a Not applicable.

b This *P* value is determined by Fisher exact test.

c N/A: not available.

**Table 2.** Medical characteristics of the study population.

Item and category	Total, n (%)	Without physical therapy, n (%)	With physical therapy, n (%)	*χ*2 (*df*)	*P* value
All patients	6090 (100)	2547 (41.8)	3543 (58.2)	—^a^	—
Chronicity risk				170.8 (2)	<.001
With indication of chronicity	506 (8.3)	109 (4.3)	397 (11.2)		
With indication of chronicity risk	1490 (24.5)	507 (19.9)	983 (27.7)		
Without indication of chronicity	4094 (67.2)	1931 (75.8)	2163 (61)		
Most common outpatient diagnosis in DiGA^b^ quarter – main categories				348.2 (33)	<.001, <.001^c^
Factors influencing health status or contact with health services (examination, investigation, and vaccination)	3704 (60.8)	1432 (56.2)	2272 (64.1)		
Musculoskeletal disorders	3139 (51.5)	1044 (41)	2095 (59.1)		
Disorders and injuries of the spine	3022 (49.6)	940 (36.9)	2082 (58.8)		
Ear, nose, and throat disorders	1894 (31.1)	753 (29.6)	1141 (32.2)		
Psychological disorders	1724 (28.3)	567 (22.3)	1157 (32.7)		
Metabolic disorders	1709 (28.1)	595 (23.4)	1114 (31.4)		
Symptoms and abnormal clinical and laboratory findings, not elsewhere classified	1708 (28)	597 (23.4)	1111 (31.4)		
Gynecological and andrological disorders	1525 (25)	555 (21.8)	970 (27.4)		
Heart diseases	1236 (20.3)	396 (15.5)	840 (23.7)		
Gastrointestinal diseases	1232 (20.2)	435 (17.1)	797 (22.5)		
Most common outpatient diagnosis in DiGA quarter – *ICD*^d^ codes				2094.7 (1044)	<.001, <.001^c^
Z01 – Examinations and investigations without complaints or reported diagnosis	2459 (40.4)	938 (36.8)	1521 (42.9)		
M54 – Dorsalgia (Back pain)	2208 (36.3)	667 (26.2)	1541 (43.5)		
I10 – Essential (primary) hypertension	1025 (16.8)	328 (12.9)	697 (19.7)		
M99 – Biomechanical lesions, not classified elsewhere	921 (15.1)	280 (11)	641 (18.1)		
M51 – Other intervertebral disc disorders	834 (13.7)	173 (6.8)	661 (18.7)		
Z30 – Contraceptive management	825 (13.5)	349 (13.7)	476 (13.4)		
Z12 – Special screening examination for neoplasms	823 (13.5)	327 (12.8)	496 (14)		
M47 – Spondylosis	768 (12.6)	204 (8)	564 (15.9)		
J06 – Acute upper respiratory infections of multiple and unspecified sites	749 (12.3)	329 (12.9)	420 (11.9)		
F45 – Somatoform disorders	692 (11.4)	158 (6.2)	534 (15.1)		
Incapacity days				38.0 (6)	<.001
None	4477 (73.5)	1920 (75.4)	2557 (72.2)		
1‐3 days	319 (5.2)	134 (5.3)	185 (5.2)		
4‐7 days	436 (7.2)	197 (7.7)	239 (6.7)		
8‐14 days	425 (7)	174 (6.8)	251 (7.1)		
15‐28 days	256 (4.2)	73 (2.9)	183 (5.2)		
29‐42 days	85 (1.4)	26 (1)	59 (1.7)		
More than 42 days	92 (1.5)	23 (0.9)	69 (1.9)		
Medication				220.6 (10)	<.001, <.001^c^
M01A – Nonsteroidal anti-inflammatory drugs (NSAIDs)	881 (14.5)	250 (9.8)	631 (17.8)		
N06A –Antidepressants	434 (7.1)	138 (5.4)	296 (8.4)		
N02B – Other analgesics and antipyretics	431 (7.1)	112 (4.4)	319 (9)		
M03B – Muscle relaxants, centrally acting agents	216 (3.5)	52 (2)	164 (4.6)		
N02A – Opioids	145 (2.4)	26 (1)	119 (3.4)		
N03A – Antiepileptics	76 (1.2)	19 (0.7)	57 (1.6)		
N05A – Antipsychotics	64 (1.1)	26 (1)	38 (1.1)		
N05C – Hypnotics and sedatives	44 (0.7)	16 (0.6)	28 (0.8)		
N01B – Local anesthetics	14 (0.2)	3 (0.1)	11 (0.3)		
M03A – Muscle relaxants, peripherally acting agents	3 (0)	0 (0)	3 (0.1)		
None of these	4489 (73.7)	2049 (80.4)	2440 (68.9)		

a Not applicable.

b DiGA: digital health application.

c This *P* value is determined by Fisher exact test

d *ICD*: *International Classification of Diseases*.

### Medical Characteristics

Among the most common diagnoses in the DiGA quarter were M54 – back pain, M99 – biomechanical dysfunction, not elsewhere classified, and M51 – other intervertebral disc disorders, as displayed in Table 2. Almost 15% (881/6090) of the patients used prescribed nonsteroidal anti-inflammatory drugs; over-the counter medications were not included in the SHI claims data. Patients with indications of chronicity or chronicity risks were significantly more likely to undergo physical therapy than those without such indications (*χ*^2^_2_=170.8; *P*<.001).

### DiGA Characteristics

The most commonly used DiGA in the study population was Vivira for back pain, followed by Companion patella and Mawendo. Most DiGAs were prescribed by physicians (5717/6090, 93.9%). The leading prescribers were orthopedic surgeons (4395/6090) with 72.2% of prescriptions, with an average of 5.2 DiGA prescriptions per health care provider. General practitioners were the second most common prescribing physicians (629/6090) with 10.3% of prescriptions. Approximately 15% (793/5065) of Vivira users continued using the DiGA beyond the intended 90-day period, opting for consecutive usage cycles, with some engaging in the same DiGA for up to a maximum of 8 consecutive quarters. Details regarding the utilization of DiGAs are presented in Table 3.

**Table 3.** Digital health applications (DiGAs) utilization.

Item and category		n/N (%)		Average number of prescriptions per physician
DiGA utilization				
Vivira		5067/6090 (83.2)		N/A^a^
Companion patella		930/6090 (15.3)		N/A
Mawendo		93/6090 (1.5)		N/A
Access to DiGA				
Prescription by a doctor or physiotherapist		5717/6090 (93.9)		N/A
Self-applications at the SHI^b^		373/6090 (6.1)		N/A
Proportion of self-applications per DiGA				
Vivira self-applications		320/6090 (5.3)		N/A
Companion patella self-applications		50/6090 (0.8)		N/A
Mawendo self-applications		3/6090 (0.1)		N/A
Prescribing physician				
Orthopedic surgeon		4395/6090 (72.2)		5.2
General practitioner or primary care internist		629/6090 (10.3)		1.8
Trauma surgeon or neurosurgeon		280/6090 (4.6)		2.3
Physiatrist		188/6090 (3.1)		4.3
Rheumatologist		122/6090 (2)		4.9
Anesthetist (including pain therapist)		37/6090 (0.6)		1.9
Other specialists		66/6090 (1.1)		1.3
N/A		373/6090 (6.1)		N/A
Vivira: quantity of DiGAs used in follow-up period per patient (follow-up prescriptions)				
Once		4272/5067 (84.3)		N/A
More than once		793/5067 (15.7)		N/A
Companion patella: quantity of DiGAs used in follow-up period per patient (follow-up prescriptions)				
Once		860/930 (92.5)		N/A
More than once		70/930 (7.5)		N/A
Mawendo: quantity of DiGAs used in follow-up period per patient (follow-up prescriptions)				
Once		92/93 (98.9)		N/A
More than once		1/93 (1.1)		N/A

a N/A: not applicable

b SHI: statutory health insurance.

### Usage Patterns of Digital and Analog Physical Therapy

**Figure 1.**
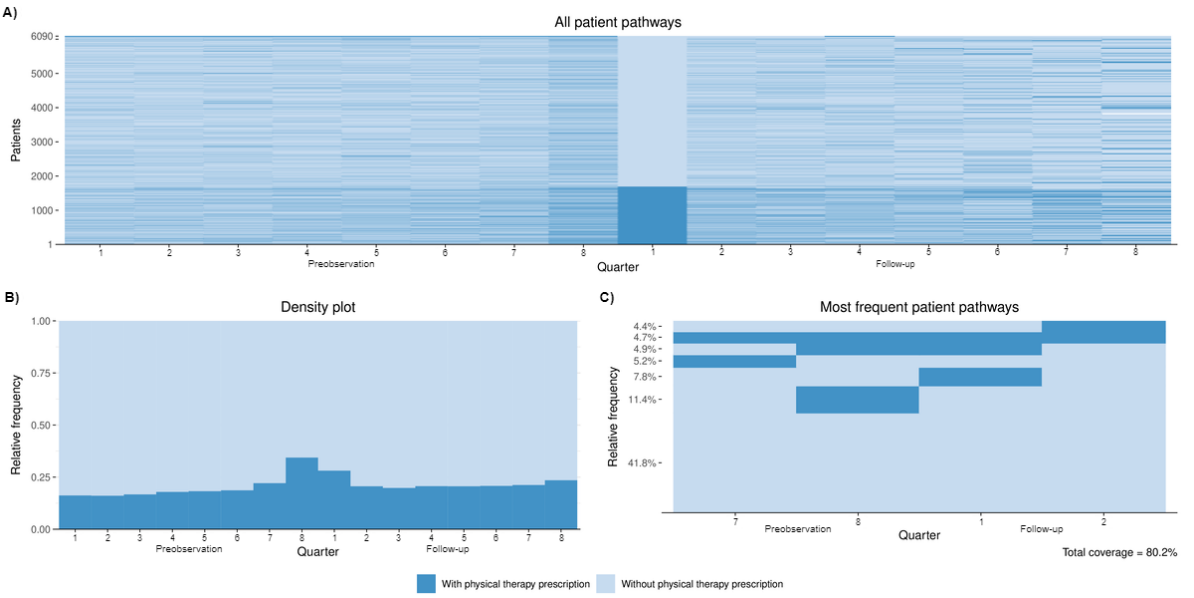
Patient pathways regarding physical therapy in addition to digital health application utilization. Quarter duration: 90 days.

During the observation period, there were noticeable alternations between quarters with and without physical therapy (see Figure 1A). Due to varying data periods, insured individuals have different lengths of follow-up periods, but at least two quarters. In the quarter of the DiGA prescription or application, 28.1% (1714/6090) of insured individuals also concurrently had a prescription for physical therapy (see Figure 1B). In most quarters, between 16.1% and 23.5% of insured persons received physical therapy, with a maximum of 34.4% (2096/6090) in the quarter leading up to DiGA use (preobservation 8). The most frequent treatment pathways indicate the concentration of physical therapy prescriptions around using the DiGA (see Figure 1C). Figure 1 provides insights into patient pathways regarding the utilization of physical therapy.

### Multivariate Analysis

Binomial logistic regression was conducted to analyze associations between chronicity, gender, age, type of DiGA, access to DiGA and the risk of using concomitant analog physical therapy. The model yielded statistically significant results (*χ*^2^_7_=380.6; *P*<.001), albeit with a relatively modest amount of explained variance, as indicated by Nagelkerke *R*^2^=.08. Notably, significant effects were observed for chronicity, gender, age and type of DiGA used. For instance, patients with indications of chronicity risk (OR 1.48 95% CI 1.31‐1.67; *P*<.001) or those with indications of chronicity (OR 2.76, 95% CI 2.22‐3.47; *P*<.001) had higher ORs to undergo physical therapy than patients without such indications. Details of all model coefficients are displayed in Table 4.

**Table 4.** Results of the binomial logistic regression model.

Variable^a^	Β (SE)	OR^b^ values (95% CI)	*P* value
Intercept	−0.88 (0.10)	0.42 (0.34-0.51)	<.001
With indication of chronicity risk	0.40 (0.06)	1.49 (1.31-1.69)	<.001
With indication of chronicity	1.02 (0.11)	2.76 (2.22-3.47)	<.001
Female gender	0.39 (0.06)	1.48 (1.33-1.66)	<.001
Age (in years)	0.02 (0.00)	1.02 (1.02-1.02)	<.001
DiGA^c^ – Mawendo	−0.47 (0.22)	0.63 (0.41-0.96)	.03
DiGA – Companion patella	−0.42 (0.07)	0.66 (0.57-0.76)	<.001
DiGA access – self-application	0.08 (0.11)	1.08 (0.87-1.35)	.50

a Variable of interest: DiGA use without physical therapy (0) versus with physical therapy (1).

b OR: odds ratio.

c DiGA: digital health application.

## Discussion

### Principal Findings

This analysis of claims data from 6090 patients with MSDs from the largest German sickness fund revealed that more than half of the DiGA users (3543/6090, 58.2%) received supplementary analog physical therapy. Orthopedic surgeons were the primary prescribers, and patients receiving DiGAs for MSDs were typically females aged between 50 and 60 years. Indicators of chronicity (risk), female gender, higher age and the DiGA used emerged as significant predictors for concomitant physical therapy use.

### Comparison With Prior Work

Our findings align with published reports, such as those of the German National Association of Statutory Health Insurances *(Spitzenverband Bund der Krankenkassen – GKV-Spitzenverband)* [17], which describe the current utilization of all DiGAs in Germany on the basis of data from over 374,000 users. In addition, the report of the TK itself includes data from over 19,000 DiGA (2022) and almost 69,000 users in 2024, and the report of the DiGA manufacturers covers data from 41 DiGA applications [20-22]. The finding that nearly 60% of the patients (3543/6090, 58.2%) received additional physical therapy is in line with a survey of 214 insured patients from another German SHI. In that publication, 54.9% reported either current use or expected use of DiGA for MSDs in addition to other health care services [26]. No further claims data studies were currently available for comparison.

The increasing adoption of DHIs, especially for managing chronic illnesses such as MSDs, highlights the need for comprehensive evaluations of their effectiveness in real-world settings. While systematic reviews have underscored the positive treatment effects of DHIs on pain and functionality [11-14], evidence regarding their long-term impact and integration into health care systems remains limited. An observational post-market study of 3629 Vivira users showed positive effects in pain reduction over 2, 4, 8, and 12 weeks in the real-world setting (T_3628_=5308; *P*<.001) especially for back pain. However, this result may be biased due to missing outcome data: 51.1% (1853/3629) of patients were lost to follow-up at 2 weeks and 87.4% (3171/3629) at 12 weeks [27]. Notably, a scoping review encompassing 87 studies demonstrated that only 17 studies (19.5%) analyzed general health care utilization of patients using DHIs [28]. Further research based on real-world data, such as in the current analysis, is warranted to complement the clinical trial-based assessment of DHIs [29,30].

### Blended Approaches

Blended therapeutic approaches integrating both digital and analog therapies offer promising evidence for optimizing health care resources and improving patient outcomes, particularly in complex cases of MSDs. These care models are relatively new to the German health care system but are evaluated in neighboring European countries. For example, a Dutch randomized controlled study of 208 patients with hip and knee osteoarthritis compared physical therapy alone (on average 12 sessions) to a hybrid of an e-exercise intervention with five face-to-face physical therapy sessions. Physical functioning and free-living physical activity were similar in both groups [31]. Although intervention costs and medication costs in the e-exercise group were significantly lower, the cost-effectiveness from the societal perspective (total costs of osteoarthritis, irrespective of the payer; eg, absenteeism and presenteeism), as well as the total health care costs (intervention costs+primary health care costs+secondary health care costs+medication costs), could not be demonstrated [32]. Blended therapy may help save health care resources, which is especially important in Germany, where provider-specific budgeting and a shortage of physical therapists can limit access to physical therapy [29].

Although the efficacy of blended therapeutic approaches appears comparable to that of analog therapy, the optimal combination of analog and digital components tailored to individual patient needs must be specified. Stepped care models, such as the one described above, which involve initiating treatment with an unguided internet-delivered intervention and subsequently extending it with physical therapist guided exercises, offer a possible approach to determine the optimal balance [31]. This strategy is supported by the findings of our claims data analysis, which showed that patients with indicators of chronicity and chronicity risk were more likely to receive physical therapy in addition to DiGA. In contrast, less complicated cases typically received DiGA as a standalone therapy. Supporting this approach, preliminary results of a multicenter, cluster-randomized controlled trial (by Koppenaal and colleagues [33]) of 208 patients with lower back pain suggest that patients with a high risk of chronicity might benefit particularly from blended therapy approaches compared to analog physical therapy. While that study did not show any difference between the treatment groups regarding physical functioning over a 3-month period, secondary outcomes such as fear-avoidance beliefs and self-reported adherence revealed significant between-group effects in favor of blended therapy [33]. Future studies need to investigate the extent to which these blended care approaches are suitable and relevant for the German health care system.

Requirements for implementing blended therapy approaches in the German health care system include the development of interfaces between digital and analog therapy, as well as a patient-centered, multidisciplinary exchange of information. Currently, for the DiGAs Vivira, Companion patella, and Mawendo, a single nonmandatory follow-up check by the prescribing physician, consisting of a PDF extraction of follow-up data from the app and a discussion with the patient, is intended. Active changes to treatment by the physician or regular provider intervention are currently not included. Additional evidence shows that feedback from health care professionals enhances self-management and adherence to DHIs, especially in musculoskeletal physical therapy [34]. Hence, the Federal Ministry of Health’s *(Bundesministerium für Gesundheit – BMG)* digitalization strategy envisages developing and establishing genuinely blended care models in the future [35].

### Strengths and Limitations

The DiGA*move* study is one of the first longitudinal real-world evidence analyses investigating DiGA utilization in comparison with conventional health care services. This analysis benefits from extensive data covering over 6000 patient trajectories in up to four years. This large sample allowed for meaningful subgroup comparisons, reflecting real-world care across various health sectors. However, our design lacked a control group of patients with MSDs who did not use DiGA. Due to the inclusion of TK-insured patients only and the inclusion criterion of using a DiGA at least one time, our sample might be biased towards tech-savvy patients who were among the first to try DiGAs in Germany. The generalizability of the findings may be limited because only three DiGAs were included, with Vivira for treating back pain being overrepresented. Incomplete data, regarding DiGA-related metrics of early prescriptions in 2020, may have impacted results.

### Conclusion

Our analysis highlights the essential role of DiGAs in comprehensive care for MSDs. Chronicity indicators emerged as significant factors influencing the concomitant use of digital and analog physical therapy, emphasizing the relevance of blended care approaches for complex cases. When developing and implementing blended care models in practical settings, interfaces between analog and digital therapies, as well as interdisciplinary collaboration among health care professionals, are necessary. Further research should identify specific patient groups that benefit most from blended therapy approaches.

## Supplementary material

10.2196/63935 Multimedia Appendix 1 Functionalities of the three digital health applications used by patients with musculoskeletal disorders in our study.

## Abbreviations

BfArM: Federal Institute for Drugs and Medical Devices (*Bundesinstitut für Arzneimittel und Medizinprodukte*)
DHI: digital health intervention
DiGA: digital health application (*Digitale Gesundheitsanwendung)*
DVG: Digitale-Versorgung-Gesetz
GPS: Good Practice in Secondary Data Analysis
MSD: musculoskeletal disorder
OR: odds ratio
SHI: statutory health insurance
STROSA: Standardisierte Berichtsroutine für Sekundärdaten Analysen
TK: Techniker Krankenkasse
