# Supplementary material for: Combined Use of Digital and Analog Physical Therapy in Patients With Musculoskeletal Disorders and Indicators of Chronicity: German Claims Data Analysis
Source: JMIR Mhealth Uhealth. 2025 Jun 9;13:e63935. doi: 10.2196/63935 (PMC12168610; doi:10.2196/63935)
Supplement: Multimedia Appendix 1 [file mhealth-v13-e63935-s001.pdf]

Multimedia Appendix 1: Functionalities of the three DiGAs used by MSDs patients in our study.

Vivira

Indication / Diagnosis	Unspecific lower back pain
Age groups	≥ 18 years of age
Intended usage period	90 days
Functionalities	1. Data entry for training program configuration (e.g. demographics, medical findings, movements impairments)<br>2. Training (4 exercises daily with continuous adaptation in intensity and complexity based on patient feedback)<br>3. Progress log and training history (e.g. pain, movement impairment, training sessions completed)<br>4. Functionality assessments (self-tests of mobility, strengths and coordination)<br>5. Educational content (knowledge articles on topics relating MSDs and the use of Vivira)<br>6. PDF progress report (core data on the therapy program, progress log, training history and treatment progress can be generated as a PDF)<br>7. Profile and settings
Provider Interaction	Optional: Information of patient, prescription (not mandatory), optional monitoring (e.g. monitoring of the PDF progress report)

companion patella powered by medi – proved by Dt. Kniegesellschaft

Indication / Diagnosis	Patella-related knee pain
Age groups	14-65 years of age
Intended usage period	90 days
Functionalities	1. Onboarding: general introduction to the program, explanations and instructions, collection of data (movement restrictions, diagnosis, etc.)<br>2. Training: 4 - 8 exercises included in the training session, video player for self-training of the exercises (based on user feedback, the exercises in the training plan can be individually adapted in terms of intensity and complexity)<br>3. Training progression: statistics on training and pain progression, improvement in knee joint function<br>4. Library: general and specific information on the clinical picture, therapy and training concept, self-help<br>5. Therapy report: users can download the key therapy data (personal data, training, pain and knee joint function history) as a PDF<br>6. Menu: general information on the manufacturer and software, privacy policy, personal data settings, consent, instructions for use, safety, support
Provider Interaction	Optional: Information of patient, prescription (not mandatory), optional monitoring (e.g. monitoring of the PDF progress report)

Mawendo

Indication / Diagnosis	Patella-related knee pain
Age groups	≥ 12 years of age
Intended usage period	12 weeks

Functionalities	1. Training with Exercise Videos and instructions: three successive exercise phases with increasing intensity and difficulty, 8-16 excercises per phase (20-40 minutes per training session, should be completed 2-3 times/week)<br>2. Information: Basic information on disorder and information on app usage<br>3. Documentation: overview page with documentation option of training duration and pain level, displayed as progression graphs<br>4. PDF reports: PDF version of training plan and PDF of training documentation and personal data
Provider Interaction	Mandatory: Initial individualization of training program ensuring specification to the diagnosis<br>Optional: Information of patient, prescription (not mandatory), optional monitoring (e.g. monitoring of the PDF progress report)

Reference:

Federal Institute for Drugs and Medical Devices. DiGA Verzeichnis [DiGA directory] 2025 URL: https://diga.bfarm.de/de/verzeichnis [accessed 2025-02-21].
